# MECHANISMS OF NEUROPROTECTION BY MTOR INHIBITORS

**DOI:** 10.1093/geroni/igz038.1350

**Published:** 2019-11-08

**Authors:** Veronica Galvan

**Affiliations:** Barshop Institute for Longevity and Aging Studies, San Antonio, Texas, United States

## Abstract

The mammalian/mechanistic target-of-rapamycin (mTOR) inhibitor rapamycin, that delays aging in mice, halts and even reverses memory deficits, and restores cerebral blood flow (CBF), neuronal activation, and neurovascular coupling in models of Alzheimer’s disease (AD), cognitive dysfunction of atherosclerosis, and normative aging. Genetic reduction of TORC1 in neurons to levels similar to those achieved by rapamycin, promoted synaptic bouton remodeling, enhanced memory, and increased brain glucose metabolism. In AD mice, the restoration of CBF and neurovascular coupling by mTOR attenuation was dependent on the activation of both constitutive nitric oxide synthase (NOS) isoforms, possibly due to stabilization of their mRNAs. The mechanisms by which mTOR attenuation preserves brain healthspan may be common to different models of age-associated neurological disease. We singled out (a) ablation of NOS activity, and (b) synaptic bouton loss as key mechanisms by which TOR drives brain aging and contributes to the pathogenesis of dementias modeled in mice.

